# A case report of septic shock syndrome caused by *S. pneumoniae* in an immunocompromised patient despite of vaccination

**DOI:** 10.1186/s12879-017-2481-y

**Published:** 2017-06-22

**Authors:** Josef Singer, Christoph Testori, Peter Schellongowski, Ammon Handisurya, Catharina Müller, Eva-Maria Reitter, Wolfgang Graninger, Paul Knöbl, Thomas Staudinger, Stefan Winkler, Florian Thalhammer

**Affiliations:** 1 Division of Infectious Diseases and Tropical Medicine, Department of Internal Medicine I, Medical University of Vienna, 1090 Vienna, Austria; 2 Department of Emergency Medicine, Medical University of Vienna, 1090 Vienna, Austria; 3 Intensive Care Unit 13i2, Department of Internal Medicine I, Medical University of Vienna, 1090 Vienna, Austria; 4 Clinical Department for Nephrology and Dialysis, Department of Internal Medicine III, Medical University of Vienna, 1090 Vienna, Austria; 5 Clinical Department of Hematology and Hemostaseology, Department of Internal Medicine I, Medical University of Vienna, 1090 Vienna, Austria

**Keywords:** Case report, *Streptococcus pneumoniae* Serovar 24F, Autoimmune lymphoproliferative syndrome (ALPS), Overwhelming post-splenectomy infection (OPSI), Vaccination

## Abstract

**Background and case presentation:**

We report a case of septic shock syndrome caused by *Streptococcus pneumoniae* in a patient who had undergone splenectomy due to an autoimmune lymphoproliferative syndrome (ALPS), which is characterized as a dysfunction of immunoregulation. Although the patient was vaccinated with a conjugated polysaccharide vaccine after the splenectomy, he was still susceptible to *S. pneumoniae* infection, because the isolated serovar (24F), a serovar long thought to be apathogenic, is not covered by any vaccine currently approved, neither a conjugated nor an unconjugated polysaccharide one.

**Conclusions:**

This case demonstrates that, due to presence of different serovars, also infections with bacteria against which patients are vaccinated have to be considered as differential diagnosis. Although vaccine development has extended the coverage of *S. pneumoniae* from 7 to 23 serovars within recent years, there is still demand for novel vaccines which can provide broader protection also against so-thought “apathogenic” strains, especially for groups at high risk.

## Background

*Streptococcus pneumoniae* is a grampositive diplococcus, which asymptomatically colonizes the upper respiratory tract [1], but can also cause diseases like rhinosinusitis [2], pneumonia [3], otitis media [4] or meningitis [5]. In severe cases *S. pneumoniae* (or “Pneumococci” as they are also termed) can induce septic shock syndrome, a life-threatening event [6]. Thus, specific prophylaxis has been developed and vaccination against the most common strains of *S. pneumoniae* is recommended for children, patients over 51 years of age and populations at risk, e.g. patients receiving immunosuppression after organ transplantation or persons with immune defects [7, 8].

As more than 90 different serovars of *S. pneumoniae* could be identified so far [9], research aims at developing vaccines that deliver broad immunity. So far, four different vaccines have been licensed and are in clinical use: Prevnar® (Wyeth/Pfizer), Synflorix® (GlaxoSmithKline), Prevnar13® (Pfizer) and Pneumovax23® (Merck) [10, 11].

Prevnar® (or Prevenar®, as it is marketed in Austria) is a heptavalent conjugated polysaccharide vaccine consisting of polysaccharides from pneumococcal serotypes 4, 6B, 9 V, 14, 18C, 19F, and 23F, individually conjugated to CRM_197_, a non-toxic diphtheria toxin mutant [12]. Synflorix® covers the same serovars as Prevnar® plus serotypes 1, 5, and 7F. It is also a conjugated polysaccharide vaccine, using nonlipidated cell-surface liporotein (protein D) of Non-Typeable Haemophilus influenzae (NTHi) as well as tetanus and diphtheria toxoid as carriers [13]. Prevnar13® (Prevenar13® in Austria), also a conjugated polysaccharide vaccine (to CRM_197_), delivers immunity against serovars 1, 3, 4, 5, 6A, 7F, 9 V, 14, 18C, 19A, 19F, 23F [14].

Pneumovax23®, the non-conjugated, 23-valent vaccine, however, consists of polysaccharides from serovars 1, 2, 3, 4, 5, 6B, 7F, 8, 9 N, 9 V, 10A, 11A, 12F, 14, 15B, 17F, 18C, 19F, 19A, 20, 22F, 23F & 33F and covers theoretically 85–90% of all circulating strains [15].

In Austria, the recommended vaccination scheme for infants is a conjugated vaccine at months 3, 5 and 12. This early start of vaccination is advised as the peak of meningitis manifestations caused by *S. pneumoniae* is within the 2nd year of life. Moreover, people over 51 years of age are advised in Austria to undergo vaccination, too. This population is not primarily affected by invasive pneumococcal diseases (meningitis or sepsis), but at risk to develop severe pneumonia. Thus, the Austrian Ministry of Health recommends a two-step immunization procedure, starting with the conjugated polysaccharide vaccine Prevenar13® followed by Pneumovax23® after 8 weeks. This serial immunization regimen has been established due to the better initial response in antibody titres to Prevenar13®, which is effectively boostered by immunization with Pneumovax23®, but also extends immunity to the 11 serovars that are not covered by Prevenar13®. It is not fully clear yet, how long these protective effects last; therefore people in Austria are advised to refresh their immunization protection. Preliminary data suggest that the protection lasts at least 3.5 years [8].

In Austria, populations at extended risk, such as patients with immune defects, HIV-positive, organ-transplanted or splenectomized patients, but also persons with cochlea implants or liquor-fistulas are advised to receive vaccinations between the age of 18 and 50 as well, depending on their pre-immunization status with either a conjugated or the non-conjugated 23-valent vaccine [8]. When and to which extent booster injections are necessary is still under investigation.

However, even an optimal vaccination status cannot fully prevent pneumococcal disease as our case report demonstrates.

## Case presentation

The 25-year old male caucasian patient suffered from an autoimmune lymphoproliferative disorder (ALPS) that was treated by splenectomy 24 months before this event. He had no previous episodes of severe infections. In March 2014, he developed malaise, chills and abdominal pain of sudden onset after heavy exercise, and contacted his nearest outpatient clinic. A routine check-up involving ECG, chest X-ray, blood pressure measurement and determination of O_2_-saturation showed no significant abnormalities. Blood results showed slightly elevated leukocyte counts with 11.3 G/L, normal red blood cell counts (4.78 T/L) and normal platelets with 294 G/L (see Table 1). The patient was sent home after transfusion of 1 L isotonic infusion solution and symptomatic therapy with metamizol and paracetamol.

**Table 1:** Blood parameters from initial presentation at the outpatient clinic of the peripheral hospital until day 5 upon admission on our intensive care unit

Parameter	1st contact at outpatient clinic	Admission to peripheral hospital	Admission to our ICU	Day 2	Day 5	Normal reference range
White blood cell count	11.3	2.8	30.3	36.47	42.7	3.9–8.8 G/L
Hemoglobin	14.5	14.1	9.7	9.6	9.0	11.9–15.4 g/dL
Platelets	294	108	33	54	31	151–304 G/L
PT	69	39	31	13	67	70–100%
aPTT	37.4	59.6	>180.0	163.2	76.5	26–36 s
Fibrinogen			72	138	432	180–350 mg/dL
D-Dimer			72.19	101.07	66.89	µg/ml
Creatinine			3.2	2.55	2.11	0.9–1.2 mg/dL
ASAT (GOT)			4278	4700	1612	<50 U/L
ALAT (GPT)			1283	1233	574	<50 U/L
Gamma-GT			39	38	28	10–71 U/L
LDH			3428	3869	999	266–500 U/L
CK			6678	15,727	11,124	10–80 U/L
Myoglobin			not determined	11,500	15,600	21–98 ng/mL
Lactate			19.9	9.6	3.7	0.63–2.44 mmol/l
CRP			8.52	11.97	24.91	<0.5 mg/dL
APACHE II-Score		23	29	22	25	
SAPS II-Score		46	59	48	51	
SOFA-Score		7	16	16	15	
DIC-Score		6	7	7	7	

Eight hours later, the general condition of the patient worsened rapidly and he had to be hospitalized because at that time he had already developed leukopenia (2.8 G/L), his platelet levels were low (108 G/L) and the serum creatinine was elevated (2.0 mg/dl). Hemostasis parameters were also altered (again depicted in Table 1). A rapid test for *S. pneumoniae*-antigen was positive and although antimicrobial therapy was started immediately, the patient developed hemodynamic problems (systolic RR: 50 mmHg) and had to be transferred to an intensive care unit (ICU). In spite of catecholaminergic therapy, fluid resuscitation and intubation, the organ dysfunction of the patient deteriorated and he developed severe septic shock with metabolic acidosis (lactate 15 mmol/L), anuric renal failure, overt disseminated intravascular coagulation (DIC) and fast spreading necrotic skin lesions, resembling purpura fulminans.

Thus, the patient received fibrinogen (Haemocomplettan®, CSL Behring) and prothrombincomplex (Beriplex®, CSL Behring) alongside clindamycin, linezolid and intravenous immunoglobulins (Pentaglobin®, Biotest Pharma GmbH) and was transferred to our intensive care unit at the Medical University of Vienna.

Upon admission, his hemodynamic and respiratory situation was stable. The metabolic acidosis, however, worsened: lactate levels increased to 18 mmol/L and signs of hepatic impairment were detectable with elevated transaminases and hypoglycemia. Moreover, creatine kinase (CK) levels were massively increased at 6678 U/L and myoglobin values were at 11,500 ng/mL. The skin lesions progressed, and confluent lesions spread over the whole face, both hands and both feet. Hemostasis parameters showed thrombocytopenia (33 G/L), a PT of 31%, an aPTT of >180 s, fibrinogen values of 72 mg/dl and massively elevated D-Dimer levels (72.19 μg/ml), resembling septic coagulopathy with purpura fulminans. According to local and international recommendations, coagulation therapy with a plasma-derived protein C concentrate (Ceprotin®, Baxalta), was initiated. An initial bolus dose of 100 U/kg body weight was given, followed by 10 U/kg/h, aiming for plasma protein C activity values of 100 U/dL. Additional hemostatic therapy consisted of fibrinogen and platelet concentrates, antithrombin replacement (aiming for antithrombin levels of 100 U/dL), and low dose heparin infusion (250 U/kg BW/h). Moreover, extracorporeal renal replacement therapy had to be started because of acute renal failure.

Table 1 summarizes the blood parameters as well as clinical scores from the initial presentation at the outpatient clinic of the peripheral hospital until day 5 upon admission on our intensive care unit.

Under this treatment the patient could be successfully stabilized. Although CK and myoglobin values continued to increase during the next 3 days and the face and the limbs of the patient were impressively hypoperfused, there were no signs for a compartment syndrome, and no surgical intervention was necessary. After peaking at day 3 upon admission, CK values decreased within the next weeks until returning into the normal range after 4 weeks. In parallel, also the hemostaseologic parameters improved and the skin situation ameliorated.

The renal situation, however, remained unchanged and until week 4 no sufficient renal function could be established. Therefore, the patient was changed from continuous hemofiltration to intermittent hemodialysis, which was well tolerated. Creatinine levels were constantly high, peaking with 7.95 mg/dl.

After 27 days, the patient could be transferred from the intensive care unit to our infectiological ward, where he stayed for another 26 days. Within this stay and under forced fluid substitution, his creatinine levels returned almost into normal range (1.27 mg/dl); also the urine output increased, so renal replacement therapy could be stopped after 4 weeks upon admission. In following, renal function returned to normal. The local skin situation improved constantly, necrotic areas were surgically removed and healed decently.

The patient could be finally transferred to a rehabilitation center, from which he was discharged fully recovered.

## Discussion

Thorough examination of initial blood cultures showed that the patient had an infection with *S. pneumoniae* serovar 24F, a serovar, which has been long thought to be apathogenic.

A recent study of the “National Reference Center for Pneumococci” at the Austrian Agency for Health and Food Safety (AGES) depicting infections with *S. pneumoniae* in 2009 revealed that 303 invasive illnesses and 19 deaths occurred due to *S. pneumoniae* in Austria. Extrapolated to the population, Austria had 3.62 invasive diseases/100,000 inhabitants and a mortality of 0.23/100,000 resulting in a lethality of 6.3% in 2009. Scaled by diagnosis 43 Meningitis- (incidence 0.52/100,000), 82 Sepsis- (0.98/100,000) and 118 pneumonia/bacteremia-diseases (1.42/100,000) were detected [16].

Scaled by serovars, the most frequent infections were caused by serovar 3 (18.66%), followed by 1 (7.84%), 14 (7.09%) 7F (7.09%), 6A (6.72%) and 4 (5.97%). All others of the 39 different identified serovars (serovars could be isolated in total in 268 cases) occurred in less than 5%. Serovar 24F, the serovar infecting our patient was identified in only 3 cases [16]. In 2011 Serovar 24F was detected in 2 cases [17] and 2012 only once [18] in Austria.

This is in line with international data, e.g. in a meta-analysis of Hausdorff et al., who surveyed articles published in MEDLINE identifying pneumococcal isolates from patients. This study revealed that serovars 1 and 14 were most frequently isolated from blood, whereas 6, 10 and 23 could be isolated most frequently from cerebrospinal fluid samples [19]. A recent study investigating pneumococcal meningitis cases in France between 2001 and 2014 revealed that among the non-vaccine covered serotypes, serovars 12F and 24F emerged after the introduction of Prevnar® and Prevnar13® [20].

Also our case clearly shows that unusual strains of *S. pneumoniae* can cause severe courses of infections, especially in immunocompromised patients. Our 25-year old patient suffers from an autoimmune lymphoproliferative disorder (ALPS), which is characterized as a dysfunction of immunoregulation [21]. In most cases, ALPS is caused by a defect of the extrinsic apoptosis pathway, which signals via the FAS-receptor [22], resulting in lymphoproliferation with clinical manifestations such as lymphadenopathy and splenomegaly [23, 24]. Our patient had to undergo repetitive extirpations of lymph nodes and was also splenectomized two years prior to the reported event, making him highly susceptible to infections by encapsuled bacteria, such as *S. pneumoniae* [25]. Especially within the first years after splenectomy, overwhelming post-splenectomy infection (OPSI) syndromes can be seen [21, 26]. As in our case, this syndrome can be associated with disseminated intravascular coagulation (DIC) and subsequent protein C deficiency. Thus, replacement therapy with plasma-derived protein C concentrates is beneficial to overcome purpura fulminans and DIC [27, 28], as it could be experimentally demonstrated that protein C is able to limit hemostatic system activation, halt DIC, and can contribute to normalization of organ microcirculation [29].

In order to prevent OPSI, our patient had been vaccinated against *S. pneumoniae*, *Haemophilus influenzae*, and *Neisseria meningitidis*. He was vaccinated with Prevenar13®, but as none of the licensed vaccines provides protection to the serovar 24F, he was still susceptible to pneumococcal infection, which thus had to be considered as differential diagnosis.

## Conclusion

Summarizing, our case demonstrates the need for continuous vaccine development. In case of *S. pneumoniae*, in recent years, novel vaccines have extended the coverage from 7 to 23 serovars, for which immunity can be delivered, but still broader protection against also so-thought “apathogenic” strains would be needed, especially for groups at high risk.

## Abbreviations

ALPS: Autoimmune lymphoproliferative disorder
OPSI: Overwhelming post-splenectomy infection
